# Hydrothermal Treatment Enhances Antioxidant Activity and Intestinal Absorption of Rutin in Tartary Buckwheat Flour Extracts

**DOI:** 10.3390/foods9010008

**Published:** 2019-12-20

**Authors:** Hye-Rin Jin, Jin Yu, Soo-Jin Choi

**Affiliations:** Division of Applied Food System, Major of Food Science & Technology, Seoul Women’s University, Seoul 01797, Korea; ckatkfkd09@swu.ac.kr (H.-R.J.); ky5031@swu.ac.kr (J.Y.)

**Keywords:** tartary buckwheat flour extract, hydrothermal treatment, antioxidant activity, cellular uptake, in vitro intestinal transport, ex vivo intestinal absorption

## Abstract

Tartary buckwheat (*Fagopyrum esculentum*) is widely used in the food industry due to its functionality, which is related to its high rutin content. However, rutin is easily converted into quercetin by an endogenous enzyme during processing, resulting in a bitter taste. In this study, rutin-enriched Tartary buckwheat flour extracts (TBFEs) were obtained by hydrothermal treatments (autoclaving, boiling, and steaming), and their antioxidant activity was evaluated in human intestinal cells. The intestinal absorption of the hydrothermally treated TBFEs was also investigated using in vitro models of intestinal barriers and an ex vivo model of intestinal absorption. The results demonstrated that all of the hydrothermally treated TBFEs had increased rutin, total polyphenol, and total flavonoid contents, which enhance the in vitro and intracellular radical scavenging activities. Antioxidant enzyme activity, cellular uptake efficiency, in vitro intestinal transport efficacy, and ex vivo intestinal absorption of the hydrothermally treated TBFEs were also enhanced compared with those of native TBFE or standard rutin. These findings suggest the promising potential of hydrothermally treated TBFEs for a wide range of applications in the functional food industry.

## 1. Introduction

Tartary buckwheat (TB, *Fagopyrum esculentum*) is widely utilized in the food industry as a major ingredient in breads, spaghetti, macaroni, noodles, and gels [1,2,3]. TB is a good source of the polyphenol compound rutin, which contributes to antioxidant, anti-inflammatory, anticancer, and antithrombotic activity [4,5,6,7]. In particular, TB has been reported to have a higher rutin content than common buckwheat (CB), at 1.67% versus 0.02% [8]. Rutin can help prevent and cure various diseases, such as hypertension, arteriosclerosis, diabetes, and obesity [9,10,11,12]. Indeed, the in vitro radical scavenging and antioxidant activities of TB milling fractions have been reported to be more pronounced than those of CB [13]. However, rutin is known to be unstable during processing [14]. Incorporation of TB flour into food formulations can convert rutin into quercetin by the endogenous rutin 3-glucosidase present in TB grains [14,15]. The temperature threshold for rutin transformation to quercetin in TB dough was investigated, demonstrating that most of its rutin was transformed to quercetin at typical processing temperatures of 25 °C, 40 °C, and 60 °C [16]. Thus, it is commonly observed that rutin content decreased, contrary to the increase in quercetin in TB-based processed food formulations [16,17,18]. Quercetin was reported to have a similar bioactivity to rutin [13,19,20]. However, TB-based processed food has a bitter taste, resulting from an elevated quercetin level compared with CB. This limits its applications in commercial foods, despite its functional benefits [21,22].

In order to prevent rutin conversion into quercetin, much attention has been given to hydrothermal treatments at 80–121 °C, which lead to the deactivation of rutin 3-glucosidase and thereby maintain high amounts of rutin in TB [16,23,24,25]. In previous research, three different hydrothermal treatments (steaming, boiling, and autoclaving) were applied to TB grains, and rutin-enriched buckwheat flours were obtained from milling fractions (hull, bran, and flour) [26]. Optical, rheological, and structural characterizations of rutin-enriched TB flours were carried out, showing rutin migration from the bran to the flour fraction upon hydrothermal treatments [26]. To date, most studies have focused on the development of hydrothermal processing to obtain rutin-enriched TB flour and its physicochemical characterization. The functionality of hydrothermally treated TB flour extract (TBFE) has not been adequately explored.

In this study, we evaluated the antioxidant activity of rutin-enriched TBFEs obtained by hydrothermal treatments (steaming, boiling, and autoclaving) in terms of radical scavenging and antioxidant enzyme activities in human intestinal cells. Comparative study with native TBFE or standard rutin was also performed. Moreover, intestinal transport efficiency of rutin-enriched TBFE was carried out using an in vitro Caco-2 monolayer, an in vitro human follicle-associated epithelium (FAE), and an ex vivo small intestinal absorption model.

## 2. Materials and Methods

### 2.1. Chemicals and Reagents

TB grains were purchased from Bongpyung-nongwon Co. Ltd. (Gangwon-do, Republic of Korea). Folin–Ciocalteu’s phenol reagent, tannic acid, sodium nitrite, aluminum chloride hexahydrate, 2,2’azinobis-(3-ethyl-benzothiazoline-6-sulfonic acid) (ABTS), potassium persulfate, hydrogen peroxide, dimethyl sulfoxide (DMSO), 4-(2-hydroxyethyl)-1-piperazineethanesulfonic acid (HEPES), ethylene glycol-bis(β-aminoethyl ether)-*N*,*N*,*N*′,*N*′-tetraacetic acid (EGTA), mannitol, sucrose, radioimmunoprecipitation assay (RIPA) buffer, skim milk, sodium bicarbonate, monosodium phosphate, and glucose were purchased from Sigma-Aldrich (St. Louis, MO, USA). Ethanol, sodium carbonate, sodium hydroxide, L-ascorbic acid, hydrogen chloride, sodium chloride, potassium chloride, calcium chloride, magnesium chloride, methanol, acetonitrile, and acetic acid were provided by Samchun Pure Chemical Co., Ltd. (Pyeongtaek, Republic of Korea). (+)-catechin, catalase (CAT) assay kit, heme oxygenase 1 (HO-1), polyclonal antibody, and β-actin polyclonal antibody, and horseradish peroxidase-conjugated anti-rabbit immunoglobulin G were purchased from ENZO Life Sciences (Farmingdale, NY, USA). 2,2-diphenyl-2-picrylhydrazyl (DPPH) and rutin trihydrate were obtained from Alfa Aesar (Ward Hill, MA, USA). Sodium dodecyl sulfate (SDS), acryl-bisacrylamide (29:1), and Tris-buffered saline-Tween 20 (TBS-T) solutions were purchased from Elpis Biotech Inc. (Daejeon, Republic of Korea). 3-(4,5-dimethyl thiazol-2-yl)-2,5-diphenyltetrazolium bromide (MTT) and H_2_DCFDA (2’,7’-dichlorofluoroescein diacetate) were provided by Duchefa Biochemie (Haarlem, Netherlands) and Molecular Probes, Inc. (Eugene, OR, USA), respectively. A superoxide dismutase (SOD) assay kit from Cayman Chemical Company (Ann Arbor, MI, USA), a protease inhibitor cocktail from Thermo Fisher Scientific, Inc. (Waltham, MA, USA), and a luminal Western blotting detection kit from Santa Cruz Biotechnology, Inc. (Santa Cruz, CA, USA) were used. Phosphate-buffered saline (PBS), minimum essential medium (MEM), heat-inactivated fetal bovine serum (FBS), penicillin, streptomycin, Roswell Park Memorial Institute (RPMI) 1640, and Dulbecco’s modified eagle’s medium (DMEM) were purchased from Welgene Inc. (Gyeongsan, Republic of Korea). Bio-Rad protein assay dye reagent and polyvinylidene fluoride (PVDF) membrane were provided by Bio-Rad Laboratories, Inc. (Hercules, CA, USA) and Merck Millipore (Burlington, MA, USA), respectively. Matrigel^®^ from Corning Inc. (Corning, NY, USA) and Transwell^®^ polycarbonate inserts from SPL Life Science Co., Ltd. (Pocheon, Gyeonggi-do, Republic of Korea) were used.

### 2.2. Extraction of Native and Hydrothermally Treated TB Flours

Hydrothermally treated TB flours (steamed, boiled, and autoclaved) were provided by Sejong University according to the method published [26]. Briefly, for steamed, boiled, and autoclaved samples, the TB grains (150 g) were steamed over boiling water in a closed chamber, soaked in boiling water (2000 mL), and autoclaved (121 °C), respectively, for 15 min. The hydrothermally treated samples were dried at 25 °C overnight, milled using a laboratory grinder (HMF-3150S, Hanil electric, Seoul, Republic of Korea), and fractionated by sieving (<150 nm). The hydrothermally treated milling fractioned flours (10 g) were extracted in 200 mL of 70% ethanol with sonication (Bransonic 5800, Branson Ultrasonics, Danbury, CT, USA) at 40 °C for 30 min as previously described [13,15]. After centrifugation (14,000× *g*, 20 min) at 4 °C, the supernatants were used for further study.

### 2.3. High-Performance Liquid Chromatography (HPLC) Analysis

Rutin content was analyzed by high-performance liquid chromatography (HPLC; Agilent 1100 series, Agilent Technologies, Santa Clara, CA, USA) equipped with a variable wavelength detector, on a Supelcosil ™ LC-18 column (250 mm × 4.6 mm i.d., 5 μm, Supelco Inc., Bellefonte, PA, USA) at 350 nm wavelength. Flow rate and injection volume were 1 mL/min and 20 μL, respectively. The mobile phase was methanol:acetonitrile:2.5% acetic acid (1:2:7, *v*/*v*/*v*).

### 2.4. Contents of Total Polyphenols and Total Flavonoids

Total polyphenol contents were measured with the Folin–Ciocalteu method [27]. TBFEs (80 μL) were mixed with 50% Folin–Ciocalteu phenol reagent (20 μL) and incubated for 5 min in the dark. After adding 2% sodium carbonate (100 μL), the solutions were further incubated for 30 min. Finally, the absorbance was measured with a microplate reader (SpectraMax^®^ M3, Molecular Devices, Sunnyvale, CA, USA) at 750 nm. The contents of total polyphenols were expressed as tannic acid equivalents (TAE g/100 g extract).

Total flavonoid contents were determined using a spectrophotometric method [28]. Briefly, 5% sodium nitrite (30 μL) was added to the TBFEs (50 μL) and incubated for 5 min. After the addition of 2% aluminum chloride hexahydrate (60 μL), the mixtures were further incubated for 6 min at room temperature in the dark. Finally, 1 N sodium hydroxide (100 μL) was added and incubated for 11 min at room temperature in the dark. The absorbance was measured at 510 nm using a microplate reader (SpectraMax^®^ M3, Molecular Devices). Catechin was used as a standard and the results were expressed as catechin equivalent (CE g/100 g extract).

### 2.5. Radical Scavenging Activity

Antioxidant activities were assessed by ABTS and DPPH assays [29,30]. ABTS radicals were produced by mixing 10 mM ABTS and 10 mM potassium persulfate with a ratio of 7.4:2.6 (*v*/*v*) at 37 °C for 24 h in the dark. After dilution with PBS (absorbance in a range of 0.65 ± 0.02 at 734 nm), ABTS radical solution (150 μL) was added to the TBFEs (50 μL), and incubation was continued for 30 min at room temperature in the dark. Finally, absorbance was measured at 734 nm using a microplate reader (SpectraMax^®^ M3, Molecular Devices).

For the DPPH assay, DPPH solution (100 μL, absorbance in a range of 0.65 ± 0.02 at 517 nm) was added to the TBFEs (100 μL) and incubated for 30 min at room temperature. Finally, the absorbance was measured at 517 nm using a microplate reader (SpectraMax^®^ M3, Molecular Devices). Antioxidant activity was expressed as L-ascorbic acid equivalent (AAE g/100 g extract).

### 2.6. Cell Culture and Cell Proliferation

Human intestinal Caco-2 cells were purchased from the Korean Cell Line Bank (KCLB, Seoul, Republic of Korea), and maintained in MEM supplemented with 10% FBS, penicillin (100 units/mL), and streptomycin (100 μg/mL) under a humidified 5% CO_2_ atmosphere at 37 °C. The effect of the extracts on cell proliferation was measured with a MTT assay. Cells (1 × 10^4^ cells/100 μL) were treated with TBFEs for 24 h. Then, 10 μL of MTT solution was added and further incubated for 4 h. Finally, 100 μL of solubilization solution (0.01 M HCl + 10% SDS) was added, and incubated overnight. The absorbance was measured at 570 nm using a microplate reader (SpectraMax^®^ M3, Molecular Devices). Concentrations that did not inhibit cell proliferation (more than 80% viability, 24-fold diluted) were used for all cell experiments (Figure S1).

### 2.7. Cellular Radical Scavenging Activity

Intracellular reactive oxygen species (ROS) were monitored with a peroxide-sensitive fluorescent probe, H_2_DCFDA [31]. Cells (1 × 10^4^ cells/100 µL) were treated with TBFEs (24-fold diluted) or standard rutin (equivalent to the rutin amount in the autoclaved sample) for 21 h and further incubated in the presence of 1 mM of hydrogen peroxide for 3 h. Next, 20 μM H_2_DCFDA was added and incubated for 30 min at 37 °C in the dark. After the addition of DMSO solution (100 μL), intracellular dichlorofluorescein fluorescence was measured using a fluorescence microplate reader (SpectraMax^®^ M3, Molecular Devices). The excitation and emission wavelengths were set to 485 and 535 nm, respectively.

### 2.8. Antioxidant Enzyme Activity

Cells (1 × 10^6^ cells/2 mL) were treated with TBFEs (24-fold diluted) or standard rutin (equivalent to the rutin amount in the autoclaved sample) for 21 h and further incubated in the presence of 1 mM of hydrogen peroxide for 3 h. The antioxidant enzyme effects of TBFEs were evaluated by measuring the activities of CAT and SOD [32,33,34], using a CAT assay kit and chemical SOD assay kit, respectively, according to the manufacturer’s protocols. Enzyme activity is presented as units U/mL.

### 2.9. Western Blot Analysis

The activity of HO-1 was estimated by Western blot analysis [35]. Cells (1 × 10^6^ cells/2 mL) were treated with TBFEs (24-fold diluted) or standard rutin (equivalent to the rutin amount in the autoclaved sample) for 21 h and further incubated in the presence of 1 mM of hydrogen peroxide for 3 h. The cells were washed with PBS and harvested using a scraper. After centrifugation (4000× *g*, 1 min) at 4 °C, cell pellets were lysed in RIPA buffer containing protease inhibitor cocktails, and incubated for 20 min with vortexing. After centrifugation at 14,000× *g* for 30 min at 4 °C, protein concentrations in cell lysates were determined using the Bradford assay.

Aliquots of lysates were subjected to 10% SDS polyacrylamide gel electrophoresis and transferred to a PVDF membrane. The blots were pre-blocked with 5% skim milk in TBS-T solution for 2 h and incubated with rabbit polyclonal antibodies against HO-1 (1:1000) and β-actin (1:5000) for 4 h. The blots were washed three times with TBS-T and incubated with horseradish peroxide-conjugated anti-rabbit immunoglobulin G (1:5000) for 2 h. Blotting analysis was performed using the luminal western blotting detection kit and a luminescent image analyzer (LAS-4000, Fujifilm, Tokyo, Japan).

### 2.10. Cellular Uptake

Cellular uptake of rutin in TBFEs was measured as described by Zhang et al. and Go et al. [36,37], with slight modification. Cells (1 × 10^6^ cells/2 mL) were incubated overnight at 37 °C and treated with TBFEs (24-fold diluted) or standard rutin (equivalent to the rutin amount in the autoclaved sample) for 24 h. After centrifugation (2500× *g*, 3 min) at 4 °C, the cell pellets were resuspended in RIPA buffer and lysed using an ultrasonic processor (Sonics & Materials Inc., Newtown, USA). After centrifugation (2500× *g*, 3 min) at 4 °C, two volumes of acetonitrile were added to the supernatants. After vortexing, the solutions were centrifuged (10,000× *g*, 3 min) at 4 °C, and the supernatants were concentrated using a nitrogen evaporator (MG-3100, Eyela, Tokyo, Japan). Finally, rutin concentrations were determined by HPLC.

### 2.11. Intestinal Transport

Human Burkitt’s lymphoma Raji B cells, supplied by KCLB, were cultured in RPMI 1640 medium, which contained FBS (10%), nonessential amino acids (1%), L-glutamine (1%), penicillin (100 units/mL), and streptomycin (100 μg/mL) in a 5% CO_2_ incubator at 37 °C. The FAE model, mimicking microfold (M) cells in Peyer’s patches, was prepared as previously described [38,39]. Briefly, Caco-2 cells (1 × 10^6^ cells/well) were cultured on upper inserts for 14 days. Raji B cells (1 × 10^6^ cells/well) in DMEM were then added to basolateral inserts and co-cultured for five days (TEER of 150–200 Ω cm^2^). Finally, the apical medium of co-culture monolayers was replaced by TBFEs (24-fold diluted) and further incubated for 2 h. The basolateral solutions were collected and used to quantify transported rutin amounts.

A Caco-2 monoculture model was used to evaluate rutin transport by the intestinal epithelial tight junction barrier. Caco-2 cells (4.5 × 10^5^ cells/well) were cultured on upper inserts for 21 days (TEER ≥ 300 Ω cm^2^); the apical medium of monolayers was replaced by TBFEs (24-fold diluted), and incubation continued for 2 h. The basolateral solutions were collected and used to quantify transported rutin amounts by HPLC. The amount of standard rutin was adjusted to be equivalent to the rutin level in the autoclaved sample.

### 2.12. Rutin Absorption in Small Intestine Sacs

Eight-week-old male SpragueDawley (200–250 g) rats were purchased from Koatech Co. (Gyeonggi-do, Republic of Korea). The animals were housed in laboratory animal cages in a ventilated room maintained at 20 °C ± 2 °C and 60% ± 10% relative humidity with a 12 h light/dark cycle. Water and commercial laboratory complete food for rats were available ad libitum. Animals were environmentally acclimated for five days before treatment. All animal experiments were performed in compliance with the guidelines issued by the Animal and Ethics Review Committee of Seoul Women’s University (SWU IACUC-2019A-13), Republic of Korea.

Everted gut sacs were prepared as previously described [40]. Briefly, two male rats were fasted overnight (with water available), and sacrificed by CO_2_ euthanasia. The small intestines were collected, washed three times with Tyrode’s solution (containing 0.8 g of sodium chloride, 0.02 g of potassium chloride, 0.02 g of calcium chloride, 0.01 g of magnesium chloride, 0.1 g of sodium bicarbonate, 0.005 g of monosodium phosphate, and 0.1 g of glucose in 100 mL of distilled water), cut into sections (5 cm in length), and everted on a puncture needle (0.8 mm in diameter). After one end was clamped, the everted sacs were filled with 200 µL of Tyrode’s solution and then tied using silk braided sutures. Each sac was placed in a six-well plate containing 3 mL of TBFEs (12-fold diluted in Tyrode’s solution) or standard rutin (equivalent to the rutin amount in the autoclaved sample) and incubated for 2 h in a humidified 5% CO_2_ atmosphere at 37 °C. The solutions in the interior sacs were collected, and the rutin content was analyzed by HPLC.

### 2.13. Statistical Analysis

Experimental data were presented as means ± standard deviations. One-way analysis of variance followed by Tukey’s test in SAS Ver.9.4 (SAS Institute Inc., Cary, NC, USA) was carried out to determine statistical significance at *P* values < 0.05.

## 3. Results and Discussion

### 3.1. Contents of Rutin, Total Polyphenols, and Total Flavonoids

**Figure 1:**
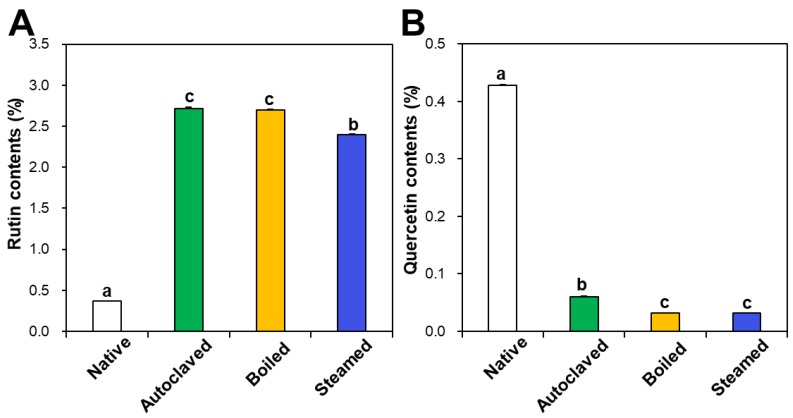
(**A**) Rutin and (**B**) quercetin contents in native and hydrothermally treated TB flour extracts (TBFEs). Different lowercase letters (a, b and c) indicate significant differences between native and hydrothermally treated (autoclaved, boiled, and steamed) extracts (*P* < 0.05).

Rutin and quercetin contents in native and hydrothermally treated TBFEs were analyzed by HPLC. Figure 1A shows that rutin concentrations increased in the hydrothermally treated TBFEs to 2.72%, 2.70%, and 2.40% in autoclaved, boiled, and steamed samples, respectively, compared to 0.37% in native TBFE. Thus, rutin levels increased by 7.35-, 7.30-, and 6.49-fold in each sample, respectively. On the other hand, quercetin contents dramatically decreased to 0.06%, 0.03%, and 0.03% in autoclaved, boiled, and steamed extracts, respectively, compared to 0.43% in native TBFE (Figure 1B). This result clearly suggests that the three hydrothermal treatments enhance rutin contents, but decrease quercetin levels in TBFEs, which is in agreement with other reports [41,42].

**Figure 2:**
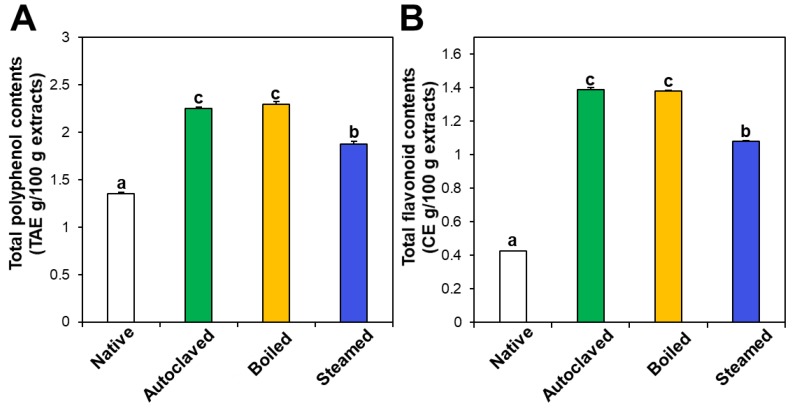
(**A**) Total polyphenol and (**B**) total flavonoid contents in native and hydrothermally treated TBFEs. Different lowercase letters (a, b and c) indicate significant differences between native and hydrothermally treated (autoclaved, boiled, and steamed) extracts (*P* < 0.05).

The contents of total polyphenols and total flavonoids also increased in the three hydrothermally treated TBFEs compared with native TBFEs (Figure 2). Statistically, the autoclaved and boiled flour extracts had higher rutin (Figure 1A), total polyphenol (Figure 2A), and total flavonoid (Figure 2B) contents than the steamed sample (*P* < 0.05). It is clear that hydrothermal treatments lead to increased rutin levels as well as total polyphenol and flavonoid contents, which play important roles in antioxidant activity. This result can be explained by rutin migration from the bran to the flour fraction upon hydrothermal treatments, showing reduced rutin in the bran, but increased rutin in the flour in the hydrothermally treated TBFEs [26]. Moreover, deactivation of rutin 3-glucosidase by hydrothermal treatments contribute to maintain high amounts of rutin, total polyphenols, and total flavonoids in TBFEs [26,41,42].

### 3.2. Radical Scavenging Activity

**Figure 3:**
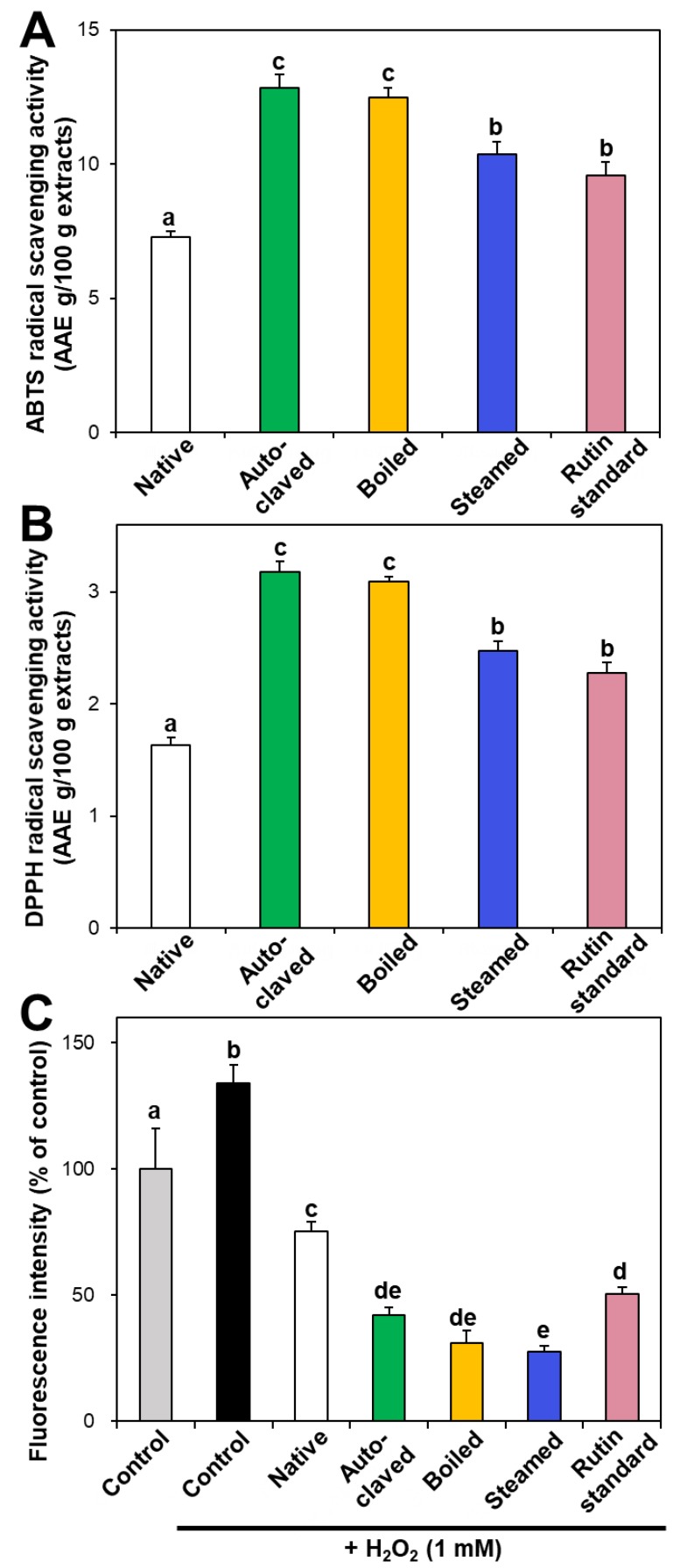
(**A**) ABTS, (**B**) DPPH, and (**C**) intracellular ROS scavenging activities of native and hydrothermally treated TBFEs. Different lowercase letters (a, b, c, d and e) indicate significant differences among control, native and hydrothermally treated (autoclaved, boiled, and steamed) extracts, and standard rutin (*P* < 0.05).

In vitro ABTS and DPPH radical scavenging activities of hydrothermally treated TBFEs were evaluated and presented in Figure 3A,B. ABTS and DPPH radical scavenging activities showed similar patterns to Figure 1 and Figure 2. Hydrothermally treated TBFEs showed higher radical scavenging activity than native TBFE. Moreover, autoclaved and boiled extracts had statistically higher activity than steamed extract. This is closely related to the rutin (Figure 1A), total polyphenol (Figure 2A), and total flavonoid (Figure 2B) contents in the first two extracts, which are higher than in the last (*P* < 0.05).

Intracellular ROS generation induced by hydrogen peroxide in human intestinal Caco-2 cells significantly decreased in the presence of native TBFE, and more dramatically decreased when hydrothermally treated TBFEs were present (Figure 3C). This clearly suggests increased ROS scavenging activity of the hydrothermally treated samples. The higher radical scavenging activity of the hydrothermally treated TBFEs than native ones must be related to the increased rutin, total polyphenol, and total flavonoid levels (Figure 1 and Figure 2). The lowest ROS generation was observed in cells incubated with steamed extract compared with autoclaved and boiled samples. It is interesting to note that significantly higher ABTS, DPPH, and intracellular ROS scavenging activities of hydrothermally treated TBFEs compared with standard rutin were found, although the amount of standard rutin was adjusted to be equivalent to the rutin level in the autoclaved sample (Figure 1A). Hence, radical scavenging activity of TBFEs seems to be not attributed to only rutin content, rather resulted from a multicomponent effect associated with rutin and other nutrients, such as various flavonoids, vitamins, and minerals [7,43,44].

### 3.3. Antioxidant Enzyme Activity

**Figure 4:**
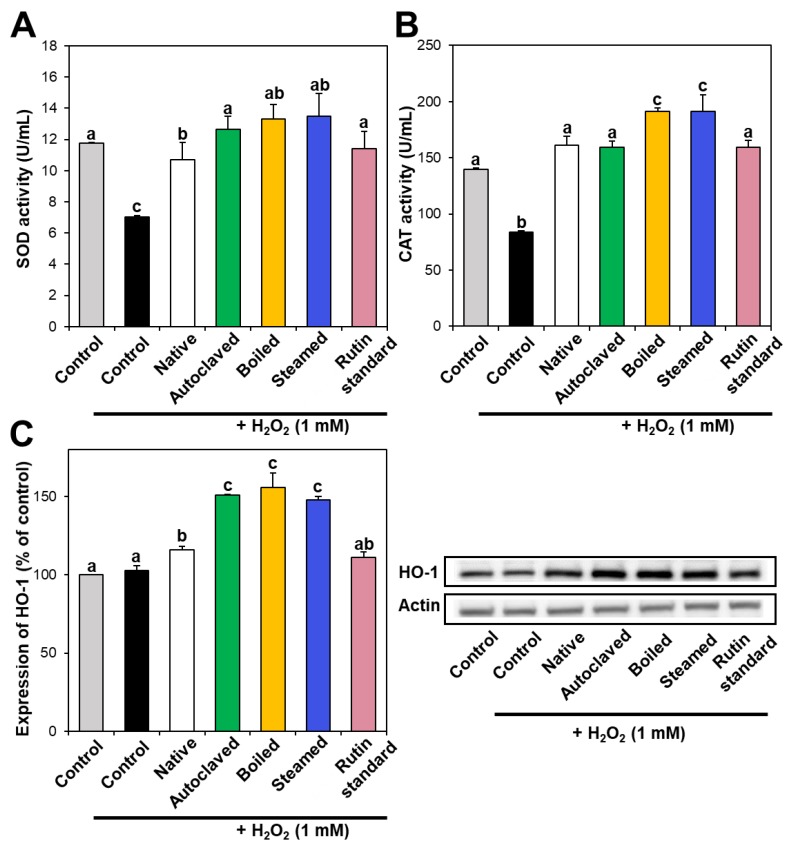
(**A**) SOD and (**B**) CAT activities and (**C**) HO-1 expression in Caco-2 cells treated with native and hydrothermally treated TBFEs. Different lowercase letters (a, b and c) indicate significant differences among control, native and hydrothermally treated (autoclaved, boiled, and steamed) extracts, and standard rutin (*P* < 0.05).

Intracellular antioxidant enzymes play a role in defense against oxidative stress, neutralizing ROS. In particular, SOD catalyzes the dismutation of the superoxide radical into oxygen or hydrogen peroxide, and CAT catalyzes the decomposition of hydrogen peroxide to water and oxygen. Figure 4A,B demonstrate that SOD and CAT activities decreased in hydrogen peroxide-treated cells, whereas they increased to the normal control levels when native TBFE was present, indicating its antioxidant activity. Moreover, SOD and CAT activities increased more remarkably in the presence of hydrothermally treated TBFEs. When the activity of HO-1, a stress-induced antioxidant enzyme, was investigated, the expression of HO-1 significantly increased in native TBFE-treated cells and more dramatically increased in the three hydrothermally treated samples (Figure 4C). These results suggest that antioxidant enzyme activity or expression increased in cells treated with TBFEs. This increase was more pronounced when the cells were incubated with hydrothermally treated TBFEs, suggesting their enhanced antioxidant effect.

It is worth noting that higher rutin, total polyphenol, and total flavonoid contents were measured in autoclaved and boiled samples than in steamed one (Figure 1 and Figure 2), which is in agreement with the results obtained by in vitro ABTS and DPPH radical scavenging activities (Figure 3A,B). Meanwhile, statistically enhanced intracellular antioxidant activity, including intracellular ROS scavenging and antioxidant CAT, SOD, and HO-1 activities, were observed in Caco-2 cells in the presence of all hydrothermally treated TBFEs (Figure 3C and Figure 4). These results suggest that increased rutin content can enhance antioxidant activity in cells, but rutin level is not the only factor affecting the antioxidant activity of TBFEs. Intracellular antioxidant functional activity of TBFEs seems to be a multicomponent combined effect resulting from rutin and other nutrients, such as flavonoids, vitamins, and minerals [7,43,44]. This is also supported by the significantly lower antioxidant activity of standard rutin, although the rutin amount was equivalent to that in the autoclaved sample (the highest level among the three hydrothermally treated TBFEs, Figure 1A). Enhanced intracellular antioxidant activity of the steamed sample, despite its relatively low rutin (Figure 1A), total polyphenol (Figure 2A), and total flavonoid (Figure 2B) amounts, may be attributed to less harsh hydrothermal condition for steaming than for autoclaving and boiling, contributing to less decomposition of other nutrients. Nevertheless, all hydrothermal treatments were found to be effective in enhancing the intracellular antioxidant activity of TBFEs. On the other hand, Zhang et al. reported that thermal treatments of TB flour, such as roasting, pressured-steam heating, and microwave heating, reduced total polyphenols, total flavonoids, and antioxidant activity [45]. This is contradictory to our results obtained by hydrothermal treatments of TB grains, which can be explained by rutin migration from the bran to the flour fraction [26]. Indeed, it was demonstrated that the bran fraction in TB had higher rutin, total polyphenols, total flavonoids, and antioxidant activity than the flour fraction [13].

### 3.4. Cellular Uptake

**Figure 5:**
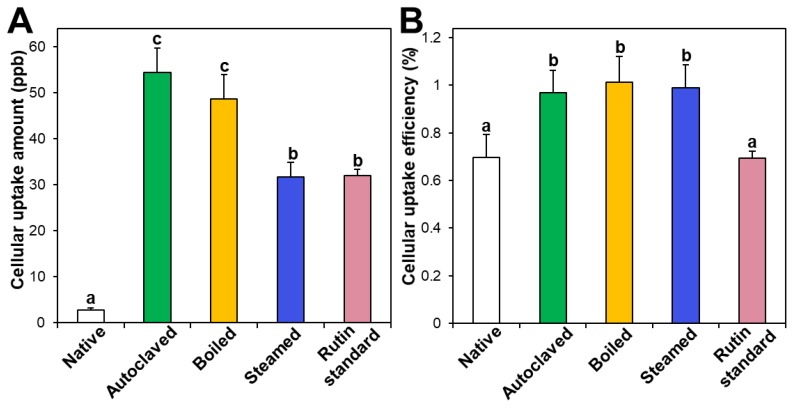
(**A**) Cellular uptake amount and (**B**) cellular uptake efficiency (%) of TBFEs and standard rutin in Caco-2 cells after incubation for 24 h. Different lowercase letters (a, b and c) indicate significant differences among native and hydrothermally treated (autoclaved, boiled, and steamed) extracts and standard rutin (*P* < 0.05).

Intestinal cellular uptake of rutin in TBFEs was evaluated after incubation for 24 h and compared with that of standard rutin. Figure 5A shows that the cellular uptake amounts of rutin in hydrothermally treated TBFEs were remarkably higher than in the native one, which is closely associated with the increased rutin concentrations (Figure 1A). Interestingly, significantly higher cellular uptake of rutin in the autoclaved sample than in standard rutin was found, although the concentration of standard rutin was the same as that in the autoclaved sample. This result suggests that the hydrothermal treatments can enhance cellular uptake in Caco-2 cells. When cellular uptake efficiency (%) was calculated, significantly higher cellular uptake efficacy of the three hydrothermally treated TBFEs was found compared with native TBFE or standard rutin (Figure 5B), supporting the role of hydrothermal treatments in enhancing cellular uptake efficiency.

### 3.5. In Vitro Intestinal Transport

**Figure 6:**
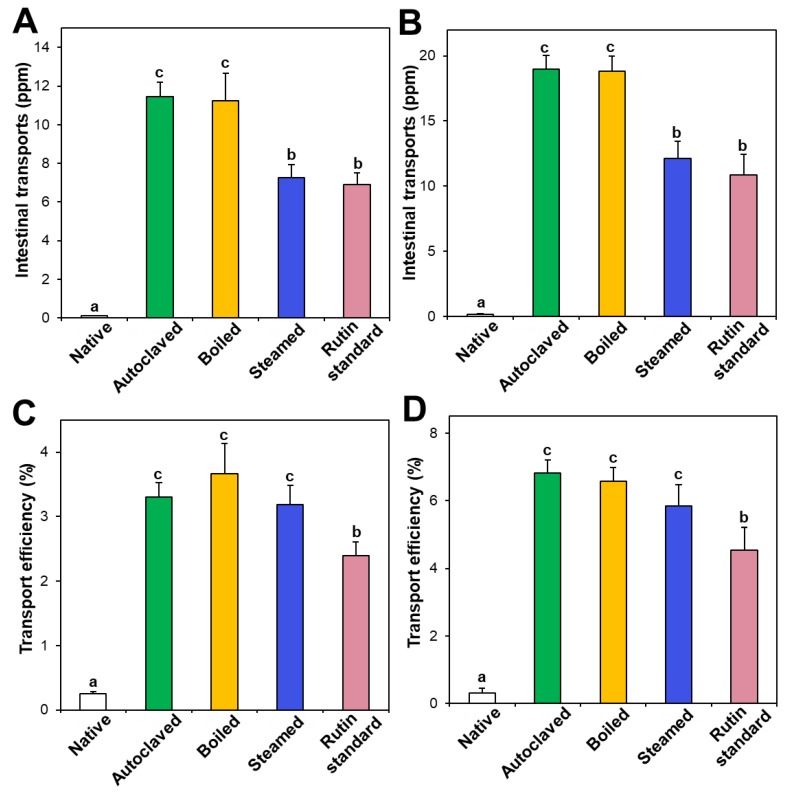
Intestinal transport (**A**,**B**) amounts and (**C**,**D**) efficiency (%) of standard rutin and rutin in native and hydrothermally treated TBFEs using in vitro models of (**A**,**C**) the Caco-2 monolayer and (**B**,**D**) FAE. Different lowercase letters (a, b and c) indicate significant differences among native and hydrothermally treated (autoclaved, boiled, and steamed) extracts, and standard rutin (*P* < 0.05).

Intestinal transport of native and hydrothermally treated TBFEs was evaluated and compared with standard rutin using in vitro models of the Caco-2 monolayer and FAE, representing intestinal tight junction, and M cells in Peyer’s patches, respectively. Figure 6A,B demonstrate that the intestinal transport amounts of hydrothermally treated TBFEs by both the Caco-2 monolayer and M cells were remarkably higher than in native TBFE, suggesting that hydrothermal treatments can increase intestinal transport amounts. Among hydrothermally treated TBFEs, higher rutin transportation of autoclaved and boiled TBFEs than the steamed extract was found, which is highly consistent with rutin levels (Figure 1A). Significantly higher transportation of hydrothermally treated TBFEs by M cells (Figure 6B) than by the Caco-2 monolayer (Figure 6A) was also found, implying that hydrothermally treated TBFEs could be primarily transported by M cells. It is interesting to note that the intestinal transport level of the autoclaved sample was significantly higher than that of standard rutin, supporting the efficiency of hydrothermal treatment for rutin transportation through intestinal barriers.

On the other hand, when transport efficiency (%) was calculated (Figure 6C,D), all the hydrothermally treated TBFEs showed higher transport efficacy (%) than standard rutin, indicating that enhanced intestinal transports of rutin in TBFEs were resulted from the hydrothermal treatments. Total intestinal transport levels, calculated by combining total amounts by both the Caco-2 monolayer and M cells, showed that the hydrothermally treated TBFEs had significantly higher transport efficacy (~10%) than that of standard rutin (~7%) or native TBFE (~0.6%).

### 3.6. Ex Vivo Intestinal Absorption

**Figure 7:**
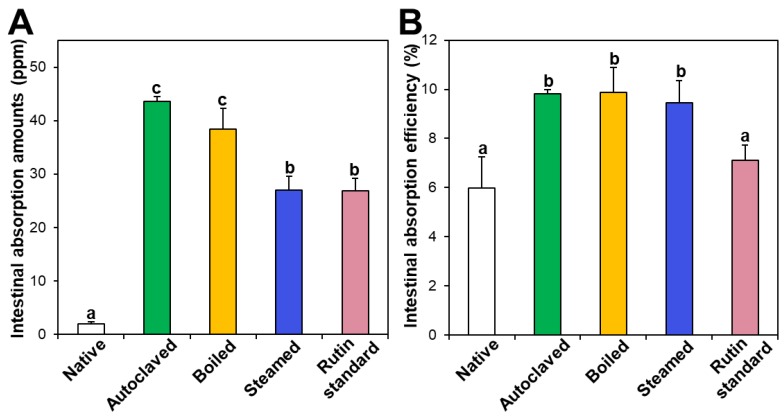
Intestinal absorption (**A**) amounts and (**B**) efficiency (%) of standard rutin and rutin in native or hydrothermally treated TBFEs using an ex vivo everted small intestine sac model. Different lowercase letters (a, b and c) indicate significant differences among native and hydrothermally treated (autoclaved, boiled, and steamed) extracts and standard rutin (*P* < 0.05).

Intestinal absorption of native and hydrothermally treated TBFEs was evaluated using an everted rat small intestine sac and compared with that of standard rutin. Figure 7A shows that the intestinal absorption amounts of all the hydrothermally treated TBFEs were remarkably higher than that of native TBFE, which attributed to increased rutin levels in the formers (Figure 1A). When the intestinal absorption levels were compared among hydrothermally treated TBFEs, the absorption levels of autoclaved and boiled samples were significantly higher than that of the steamed sample. This result is in agreement with the higher rutin contents of the two former extracts than that of the latter (Figure 1A). It is interesting to note that the intestinal absorption of standard rutin was significantly lower than that of autoclaved TBFE, although the rutin concentrations were the same.

On the other hand, when intestinal absorption efficiency (%) was calculated, all three hydrothermally treated TBFEs showed higher absorption efficacy than native TBFE, without significant differences among the hydrothermally treated TBFEs (Figure 7B). Significantly lower absorption efficiency (%) of standard rutin than hydrothermally treated TBFEs was also found. It should be noted that the intestinal transport evaluated using in vitro models of the Caco-2 monolayer and FAE (Figure 6) was highly consistent with the result obtained using an everted gut sac model (Figure 7). All the results suggest that hydrothermal treatments can enhance the intestinal absorption efficiency of rutin in TBFE.

## 4. Conclusions

The antioxidant activity and intestinal absorption efficiency of the hydrothermally (autoclaving, boiling, and steaming) treated TBFEs were evaluated in human intestinal Caco-2 cells, in vitro models of intestinal barriers, and an ex vivo everted gut sac model and compared with those of native TBFE or standard rutin. The results demonstrate that rutin, total polyphenol, and total flavonoid contents in TBFEs significantly increased by all hydrothermal treatments, contributing to significantly enhance in vitro radical and intracellular ROS scavenging activity, antioxidant enzyme activity, cellular uptake efficiency, intestinal transport efficacy, and ex vivo intestinal absorption compared with native TBFE. The antioxidant activity and intestinal absorption of the hydrothermally treated TBFEs were more pronounced than those of standard rutin, suggesting the role of hydrothermal treatments in enhanced functionality of TBFEs. It is, therefore, concluded that hydrothermally treated TBFEs are promising for various applications in the functional food industry.

## Supplementary Materials

The following are available online at https://www.mdpi.com/2304-8158/9/1/8/s1, Figure S1: Cell proliferation of TBFEs (24-fold diluted) on Caco-2 cells after incubation for 24 h. No significant differences were found among untreated control, native, and hydrothermally treated extracts (*P* > 0.05).
