# Supplementary material for: Hydrothermal Treatment Enhances Antioxidant Activity and Intestinal Absorption of Rutin in Tartary Buckwheat Flour Extracts
Source: Foods. 2019 Dec 20;9(1):8. doi: 10.3390/foods9010008 (PMC7022688; doi:10.3390/foods9010008)
Supplement: Supplementary file 1 [file foods-09-00008-s001.pdf]

Article

Hydrothermal Treatment Enhances Antioxidant Activity and Intestinal Absorption of Rutin in Tartary Buckwheat Flour Extracts

Hye-Rin Jin, Jin Yu and Soo-Jin Choi *

Division of Applied Food System, Major of Food Science & Technology, Seoul Women's University, Seoul 01797, Republic of Korea; ckatkfkd09@swu.ac.kr (H.-R.J.); ky5031@swu.ac.kr (J.Y.)

* Correspondence: sjchoi@swu.ac.kr (S.-J.C); Tel.: +82-2-970-5634; Fax: +82-2-970-5977

Received: 29 November 2019; Accepted: 18 December 2019; Published: date

Supplementary materials

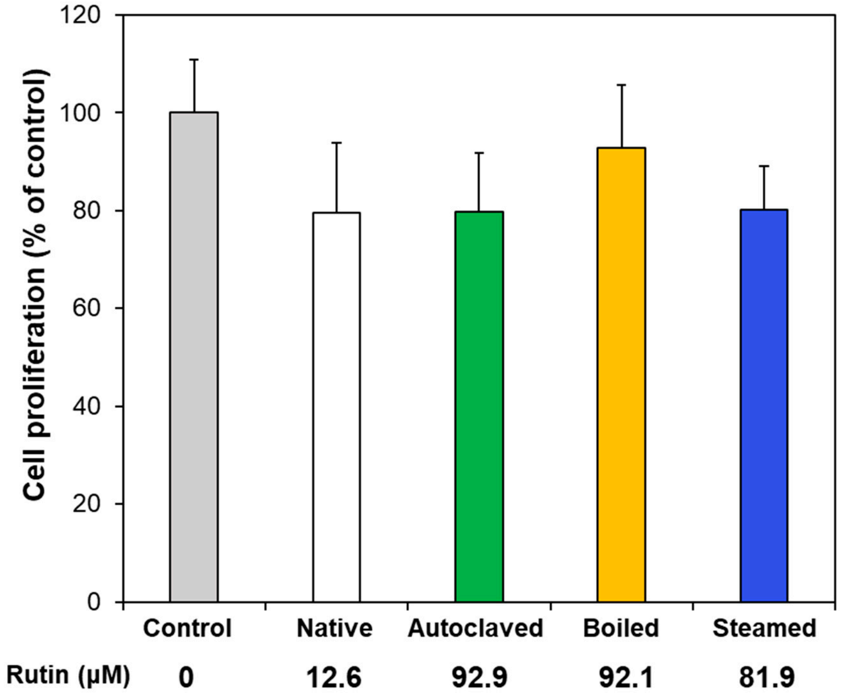

Figure S1. Cell proliferation of TBFEs (24-fold diluted) on Caco-2 cells after incubation for 24 h. No significant differences were found among untreated control, native, and hydrothermally treated extracts (P>0.05).

© 2019 by the authors. Submitted for possible open access publication under the terms and conditions of the Creative Commons Attribution (CC BY) license (http://creativecommons.org/licenses/by/4.0/).
